# Analysis of heparanase isoforms and cathepsin B in the plasma of patients with gastrointestinal carcinomas: analytical cross-sectional study: Análises das isoformas de heparanase e da catepsina B em plasma de pacientes com carcinomas gastrointestinais: estudo transversal analítico

**DOI:** 10.1590/1516-3180.2013.7080003

**Published:** 2014-10-28

**Authors:** Carina Mucciolo Melo, Clarice Silvia Taemi Origassa, Thérèse Rachell Theodoro, Leandro Luongo Matos, Thaís Aguilar Miranda, Camila Melo Accardo, Rodrigo Ippolito Bouças, Eloah Rabello Suarez, Madalena Maria Nunes Silva Pares, Daniel Reis Waisberg, Giovanna Canato Toloi, Helena Bonciani Nader, Jaques Waisberg, Maria Aparecida Silva Pinhal

**Affiliations:** I MSc. Doctoral Student, Department of Biochemistry, Universidade Federal de São Paulo (Unifesp), São Paulo, Brazil.; II MSc. Doctoral Student, Faculdade de Medicina do ABC (FMABC), Santo André, São Paulo, Brazil.; III MD, PhD. Associate Professor, Department of Biochemistry, Faculdade de Medicina do ABC (FMABC), Santo André, São Paulo, Brazil.; IV MSc. Research Collaborator, Department of Biochemistry, Universidade Federal de São Paulo (Unifesp), São Paulo, Brazil.; V PhD. Postdoctoral Student, Department of Biochemistry, Universidade Federal de São Paulo (Unifesp), São Paulo, Brazil.; VI PhD. Research Collaborator, Department of Biochemistry, Universidade Federal de São Paulo (Unifesp), São Paulo, Brazil.; VII PhD. Research Collaborator, Associação Beneficente de Coleta de Sangue (Colsan), São Paulo, São Paulo, Brazil.; VIII MD. Attending Physician. Surgery Department, Universidade de São Paulo (USP), São Paulo, Brazil.; IX Medical Student. Faculdade de Medicina do ABC (FMABC), Santo André, São Paulo, Brazil.; X PhD. Titular Professor, Department of Biochemistry, Universidade Federal de São Paulo (Unifesp), São Paulo, Brazil.; XI MD, PhD. Adjunct Professor, Department of Surgery, Faculdade de Medicina do ABC (FMABC), Santo André, São Paulo, Brazil.; XII PhD. Titular Professor, Department of Biochemistry, Faculdade de Medicina do ABC (FMABC), Santo André, São Paulo, Brazil.

**Keywords:** Glucuronidase, Cathepsin B, Tumor markers, biological, Carcinoma, Plasma

## Abstract

**CONTEXT AND OBJECTIVE:**

Heparanase-1 degrades heparan sulfate and has been correlated with tumor progression. Although the isoform heparanase-2 has no catalytic activity, it seems to be important for modulating heparanase-1 activity. Cathepsin B is a proteinase involved in tumor metastasis. The aim of this study was to analyze heparanase isoform expression and cathepsin B activity in plasma samples from patients with gastrointestinal carcinomas, compared with healthy individuals (control group).

**DESIGN AND SETTING:**

This was an analytical cross-sectional study. Peripheral blood samples were collected at a Brazilian public hospital, from 21 patients with histopathological diagnoses of gastrointestinal carcinomas and from 43 healthy individuals. The analyses were performed in two Brazilian medical schools.

**METHODS:**

Heparanase isoforms were identified and quantified in plasma samples by means of Western blot. The enzymatic activities of heparanase-1 and cathepsin B were also measured.

**RESULTS:**

The results demonstrated that the expression of both heparanase isoforms was significantly greater in plasma samples from gastrointestinal carcinoma patients, compared with the control group. Logistic regression analysis showed that increased heparanase-1 and heparanase-2 expression was exclusively dependent on the tumor. There was a significant increase in heparanase-1 and cathepsin B activity in the patients’ plasma.

**CONCLUSION:**

Overexpression of heparanase-1 and heparanase-2, along with increased heparanase-1 and cathepsin B activity in plasma, is associated with the diagnosis of gastrointestinal carcinoma. These findings provide support for using non-invasive assays (plasma samples) as an auxiliary method for diagnosing gastrointestinal tumors.

## RESUMO

**CONTEXTO E OBJETIVO:**

A heparanase-1 degrada heparam sulfato e está relacionada à progressão de tumor. Apesar de a isoforma heparanase-2 não possuir atividade catalítica, parece ser importante para modular a atividade da heparanase-1. A catepsina B é uma proteinase envolvida na metástase de tumores. O objetivo deste estudo foi analisar a expressão das isoformas de heparanase e atividade da catepsina B em amostras de plasma de pacientes com carcinomas gastrointestinais, comparando-se com indivíduos saudáveis (grupo controle).

**TIPO DE ESTUDO E LOCAL:**

Este é um estudo transversal analítico. Foram coletadas amostras de sangue periférico, em hospital público brasileiro, de 21 pacientes com diagnóstico histopatológico de carcinoma gastrointestinal e 43 indivíduos saudáveis. As análises foram realizadas em duas faculdades de medicina brasileiras.

**MÉTODOS:**

As isoformas da heparanase foram identificadas e quantificadas em amostras de plasma por Western blot. As atividades enzimáticas de heparanase-1 e catepsina B foram também mensuradas.

**RESULTADOS:**

Os resultados demonstraram que as expressões das isoformas de heparanase foram significativamente maiores nas amostras de plasma de pacientes com carcinoma gastrointestinal em comparação com o grupo controle. A análise feita por regressão logística mostra que aumento na expressão de heparanase-1 e heparanase-2 é exclusivamente dependente da presença de tumor. Houve aumento significativo na atividade da heparanase-1 e catepsina B no plasma dos pacientes.

**CONCLUSÃO:**

A superexpressão de heparanase-1 e heparanase-2, bem como o aumento da atividade de heparanase-1 e catepsina B no plasma, está associada com o diagnóstico de carcinoma gastrointestinal. Tais achados dão suporte ao uso de ensaios não invasivos (amostras de plasma) como método auxiliar para o diagnóstico de tumores gastrointestinais.

## INTRODUCTION

Gastrointestinal carcinomas remain one of the leading causes of cancer-related death worldwide.^1^ The major cause of mortality among patients with different gastrointestinal cancers is distant metastasis, rather than the primary carcinoma itself.

The incidence of hepatocarcinoma ranges from 1 to 9.34 cases per 100,000 inhabitants, depending on the region of Brazil.^2^ This type of cancer presents very fast development and therefore an early diagnosis is important for successful treatment.

In 2008, one million new cases of gastric neoplasms were expected. Gastric cancer has the fourth highest incidence in the world, and a high mortality rate.^3^

Duodenal papillary carcinoma is an extremely rare type of cancer and it is generally diagnosed by means of biopsy.^4^ In the United States, the incidence rate of duodenal papillary carcinoma is less than 5% of all cases of digestive neoplasms.^5^

In 2013, 102,480 new cases of colon cancer and 40,340 of rectal carcinoma were recorded. Colorectal carcinomas are usually diagnosed by means of biopsy.^6^

Esophageal cancer is a relatively uncommon malignancy and is an extremely lethal tumor. In Brazil, during 2005, the expected incidence was 8.99 and 2.61 new cases of esophageal cancer per 100,000 men or women, respectively.^7^

Invasion of malignant cells involves interaction with the extracellular matrix and the basement membranes. Basement membranes consist essentially of collagen type IV, laminin and heparan sulfate proteoglycans. Tumor cells need to degrade basement membrane constituents in order to promote metastasis, and this activity of malignant cells involves proteinases and other enzymes such as heparanase.^8^

Heparanase is an endo-beta-glucuronidase that degrades heparan sulfate chains of proteoglycans and has a multifunctional modulatory effect on cancer cell progression and cell-extracellular matrix interaction.^9^^,^^10^^,^^11^ Research has shown that heparanase has important roles in relation to the progression of esophageal, stomach and colonic cancer. Heparanase expression has been closely correlated with the diagnosis/prognosis of gastrointestinal cancer. Heparanase silencing can be useful as a potential anticancer therapy.^12^

There are two members of the heparanase family: heparanase-1 (HPSE1) and heparanase-2 (HPSE2), which are encoded by different genes: 4q21.3 and 10q23.24, respectively.^13^^,^^14^^,^^15^^,^^16^^,^^17^ The two isoforms of heparanase present different tissue distributions and different cell locations. Immunofluorescence and cell fractionation studies have shown that heparanase-1 is present in the nucleus, perinuclear region and plasma membrane.^18^^,^^19^

It is well known that heparanase-1 shows a direct correlation with tumor metastasis. Heparanase-1 presents two isoforms: firstly, a precursor with no apparent enzymatic activity (65 kDa), which then undergoes proteolytic activity to form the mature active enzyme, which consists of a heterodimer containing a 50 kDa subunit in association with an 8 kDa subunit.^9^^,^^20^

Nevertheless, more studies need to be conducted to clarify the relative contribution of heparanase-2 to human health and disease, since heparanase-2 does not present enzymatic activity.^15^^,^^21^ Heparanase-2 has three alternative variant splice transcripts (heparanase-2a, heparanase-2b, and heparanase-2c), which encode the putative proteins of 480, 534, and 592 amino acids, respectively. It has been predicted from sequence analysis that all three heparanase-2 proteins are intracellular, membrane-bound proteins.^15^

Alignment of the predicted coding region of heparanase-2a to heparanase-1 has revealed that the two proteins have overall identicalness of 35%.^15^ There is some evidence that heparanase-2 attenuates heparanase-1 activity, possibly conferring a favorable outcome upon head and neck cancer patients.^21^

Cathepsin B is a lysosomal cysteine proteinase, which has been implicated in a variety of diseases, such as inflammation and tumor metastasis. Overexpression of cathepsin B has been observed in malignant tumors, and specifically in the cells at the invasive edge of these tumors. Cathepsin B may facilitate invasion directly by degrading extracellular matrix components or indirectly by activating other proteases capable of digesting the extracellular matrix.^22^

Many studies have been conducted using tissue samples to investigate the expression of cathepsin B and heparanase isoforms in a variety of cancers. The present study had the advantage of using a noninvasive assay, using plasma samples.

## OBJECTIVE

The aim of this study was to evaluate isoforms of heparanase and cathepsin B in plasma samples from patients with primary gastrointestinal carcinoma, in comparison with healthy individuals.

## METHODS

### Study design and ethics

This was an analytical cross-sectional study that aimed to evaluate heparanase isoforms and cathepsin B in plasma samples from patients with different gastrointestinal carcinomas, in comparison with a control group (healthy individuals).

This study was conducted in accordance with the ethical principles of the Declaration of Helsinki. Peripheral blood samples were collected from 21 patients with histologically confirmed gastrointestinal carcinoma and from 43 healthy volunteers with no evidence of malignant tumors or inflammatory intestinal disease. The blood was collected after informed consent had been obtained. This study conformed to the regulations of the Human Ethics Research Committee of the ABC Medical School (Santo André, Brazil), under number 025/2008, and the Human Ethics Research Committee of the Servidor Público Estadual Hospital (São Paulo, Brazil), under number 021/08.

### Settings

The samples were collected in a Brazilian public hospital that provides primary, secondary and tertiary care. This study was conducted in two different Brazilian medical schools.

All patients who had been admitted for surgical treatment over the period from January to March 2009 and who fulfilled the eligibility criteria were selected. The control group was composed of adult volunteers of both genders without any evidence or suspicion of malign gastrointestinal neoplasia, pre-neoplasic lesions or any inflammatory intestinal disease, who were recruited between January and March 2009.

### Patients and clinical features

The eligibility criteria were as follows: age ≥18 years; histopathologically confirmed diagnosis of gastrointestinal cancer; no prior chemotherapy or radiotherapy; no prior treatment for other cancer; and no associated acute disease. The patient group included 21 individuals who had undergone operative procedures in order to remove primary gastrointestinal cancer, while 43 healthy individuals constituted the control group.

The patient group included 16 men (76.2%) and 5 women (23.8%). The average age in the patient group was 68.7 ± 10.0 years (ranging from 50 to 82 years). All the patients were Caucasian. The control group included 32 men (74.4%) and 11 women (25.6%) with an average age of 52.9 ± 5.0 years (ranging from 46 to 65 years).

The carcinomas were located in the stomach in 5 patients (23.8%), colon in 5 (23.8%), esophagus in 4 (19.0%), rectum in 4 (19.0%), duodenal papilla in 2 (9.5%) and liver in 1 (4.8%). Concerning histological differentiation, 4 lesions (19.0%) were poorly differentiated, 10 (47.6%) were moderately differentiated and 5 (23.8%) were well differentiated. The lymph nodes were compromised by the tumor in 14 patients (66.6%) and were disease-free in 7 (33.3%). Venous invasion occurred in 4 patients (19.0%), lymphatic invasion occurred in 3 (14.3%) and neural infiltration was present in 2 (9.5%). Among these 21 patients, distant metastasis was revealed in 7 (33.3%). Four patients (19.0%) died due to metastasis or local recurrence from carcinoma.

### Blood sample collection

Peripheral blood samples were collected using ethylenediamine tetraacetic acid (EDTA), as an anticoagulant. Sodium citrate could not be used as an anticoagulant, because of platelet activation and the release of heparanase-rich platelet-dense granules in plasma samples. Plasma samples were obtained after peripheral blood centrifugation (1,500 x g, 15 min and 4 °C). The plasma samples were frozen in small aliquots and were thawed just once for the analysis.^23^^,^^24^

### Western blot analysis

For immunoblot analysis, 2 or 5 µg of total plasma protein aliquots were loaded for electrophoresis on 10% sodium dodecyl sulfate-polyacrylamide gel (SDS-PAGE), for heparanase-1 and heparanase-2 detection, respectively. It is important to note that more than twice the amount of total plasma protein was used to analyze heparanase-2 isoforms in the Western blot assays compared with the amount used for heparanase-1 proteins. The proteins were transferred to an Immobilon-P membrane (Millipore, Bedford, Massachusetts, USA), followed by successive incubations with block solution (1% nonfat milk) containing anti-heparanase polyclonal antibody (anti-HPA) H-80 (Santa Cruz Biotechnology, Santa Cruz, California, USA) and anti-HPA C-17 (Santa Cruz Biotechnology, Santa Cruz, California, USA), for heparanase-1 and heparanase-2 analysis, respectively. The primary anti-heparanase antibodies were diluted 1:100 in 0.1% bovine serum albumin, 10 mmol/L Tris-HCl (pH 7.5), 100 mmol/L NaCl and 0.05% Tween-20. The secondary antibody was horseradish peroxidase-conjugated anti-rabbit/mouse antibody (Jackson Laboratories, Bar Harbor, Maine, USA). The immunoblot reaction was detected using 0.5% 3,3’-diaminobenzidine (DAB) and 0.01% H_2_O_2_. A mixture of standard protein markers (Sigma Chemical Co., St. Louis, Missouri, USA) was used for relative molecular mass determination. The Western blot quantification was performed by means of densitometry analysis using Scion Image software, version 4.03 (Scion Corporation, Frederick, Maryland, USA).

### Quantification of the product obtained through heparanase

The heparanase activity level was determined in terms of the heparan sulfate products generated through enzymatic activity, using 15% biotinylated heparan sulfate as the substrate, immobilized in a poly-L-lysine multi-well. The assay was performed using an Enzyme-Linked Immunosorbent Assay (ELISA)-like method.^25^ The enzymatic assay was performed in sodium acetate buffer (25 mM, pH 5.0). Diluted plasma (1:10) was incubated with biotinylated heparan sulfate on a pre-coated plate overnight, with a final volume of 100 µL. After several washings, non-degraded biotinylated heparan sulfate was detected by means of incubation with europium-conjugated streptavidin (1:1.000), for 30 min, at 25 ºC. The plate was washed five times with acetate buffer to remove unbound streptavidin. Finally, enhancement solution (Delfia, Wallac Oy, Turku, Finland) was added and the plate was shaken for 10 min. This procedure released europium that had been bound to streptavidin. A time-resolved fluorometer was used to measure free europium levels and the data (expressed as count/s) was processed automatically, using MultiCalc software (PerkinElmer Life Sciences-Wallac Oy, Turku, Finland). A standard curve of different concentrations of biotinylated heparan sulfate was produced and the result was determined as the percentage of degraded heparan sulfate.

### Cathepsin B assay

The cathepsin B enzymatic action was measured using a spectrofluorometer, using the fluorogenic substrate carbobenzoxy-Phe-Arg-7-amide-4-methylcoumarin (Z-FR-MCA, Sigma-Aldrich), as described previously.^26^ The incubations were carried out on dark microplates (Nunc, Sigma-Aldrich), at 37 ºC in 50 mM sodium phosphate buffer (pH 6.3), containing 200 mM NaCl (Millipore) and 2 mM EDTA (Sigma-Aldrich). The fluorescence intensity was measured in a microplate reader (FLEXStation 3) with the SoftMax software (Molecular Devices), with the excitation and emission wavelengths set as 360 and 465 nm, respectively. The assay was performed initially by pre-incubating the plasma samples with the enzyme activator in 2 mM of dithiothreitol (DTT, Sigma-Aldrich), for 20 minutes at room temperature, and then 5 µM of the irreversible inhibitor E-64 (Sigma-Aldrich) and the substrate (10 µM) were added. Thus, the enzymatic assay was performed for 24 hours, at 37 ºC. The level of cathepsin B action was determined in terms of substrate degradation, expressed as arbitrary units of fluorescence (AUF).

### Statistical analysis

Statistical analysis was performed using the SPSS 16.0 software for Windows (SPSS, Chicago, Illinois, USA). The Kolmogorov-Smirnov test was used to analyze whether the data had normal distribution, and the study variables were considered to be parametric. Subsequently, statistical differences among the experimental groups were evaluated using Student’s t test and the Mann-Whitney U-test. The Pearson correlation was analyzed to evaluate any possible interconnection between patients’ ages and the results obtained. Logistic regression analysis was performed to investigate whether the levels of heparanase isoforms and cathepsin B alterations were dependent on the presence of a tumor. The significance level was set at P < 0.05.

## RESULTS

### Protein expression of heparanase isoforms in the plasma

**Figure 1.**
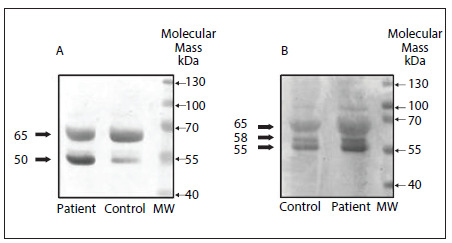
Heparanase isoform detection by means of Western blot on plasma samples. (A) represents heparanase-1: both isoforms of heparanase-1, i.e. 65 kDa (precursor) and 50 kDa (active fraction). (B) represents heparanase-2: three isoforms of heparanase-2 comprising respectively 65 kDa, 58 kDa and 55 kDa. Patient and Control correspond to plasma samples from patients with gastrointestinal carcinoma and plasma samples from healthy individuals, respectively. MW refers to standard protein markers.

Figure 1 shows heparanase-1 and heparanase-2 protein detection in the plasma of gastrointestinal patients and healthy individuals (controls), through analysis using Western blot.

Two isoforms of heparanase-1 (50 kDa and 65 kDa) can be observed in Figure 1A. It was also demonstrated that the heparanase-1 levels in the plasma of gastrointestinal carcinoma patients are higher than in the plasma of the control group (Figure 1A).

The polyclonal antibody anti-heparanase-2 C-17, which was used in the Western blot analysis, was able to identify three isoforms containing 480, 534 and 592 amino acids, named respectively, heparanase-2a, heparanase-2b and heparanase-2c. The levels of all heparanase-2 isoforms were higher in the plasma of patients with gastrointestinal carcinoma than in the control group, as shown in Figure 1B.

Quantitative analysis from Western blot assay was performed for each heparanase isoform, i.e. for heparanase-1 (50 kDa), heparanase-1 (65 kDa) and heparanase-2, as shown in Table 1. The protein expression levels in the plasma of the gastrointestinal carcinoma patients was significantly higher than in the plasma of the healthy individuals for all heparanase isoforms (Table 1).

**Table 1.**
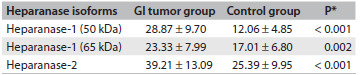
Quantitative analysis of heparanase-1 and heparanase-2 in the plasma of patients with gastrointestinal (GI) tumors and healthy controls, expressed as mean ± standard deviation of pixels/µg of total plasma protein

*Student’s t test.

The active form of heparanase-1 (50 kDa) presented higher protein expression in the gastrointestinal carcinoma patients than in the control group: respectively, 28.87 ± 9.70 pixels/µg of total protein and 12.06 ± 4.85 pixels/µg of total protein. The data showed a significant difference in the expression of plasma heparanase-1 (50 kDa) (P < 0.001; Student’s t test), as demonstrated in Table 1.

Similar results were obtained for pro-enzyme heparanase-1 (65 kDa), which demonstrated plasma expression of 17.01 ± 6.80 pixels/µg of total protein in the control group, whereas higher protein expression (23.33 ± 7.99 pixels/µg of total protein) was observed in the plasma of the gastrointestinal carcinoma patients (P = 0.002; Student’s t test), as shown in Table 1.

The average heparanase-2 expression in the plasma of the patients with gastrointestinal carcinoma was significantly greater (39.21 ± 13.09 pixels/µg of total protein) than in the control group (25.39 ± 9.95 pixels/µg of total protein), as shown in Table 1 (P < 0.001; Student’s t test).

Furthermore, patients were grouped according to their gastrointestinal tumor types and the heparanase isoforms were quantitatively analyzed in each subgroup, in relation to the control group (Table 2). These results demonstrated that the level of the active fraction of heparanase-1 (50 kDa) was significantly higher in all tumor subtypes, compared with the control group (Table 2). Moreover, heparanase-2 protein expression was significantly higher in all subtypes of gastrointestinal carcinomas.

**Table 2.**
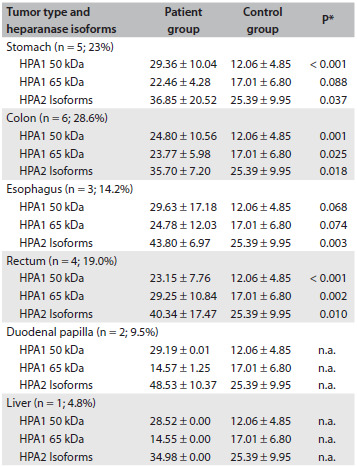
Quantitative HPA1 and HPA2 analysis on the plasma of different gastrointestinal carcinoma types expressed as mean ± standard deviation of pixels/µg of total plasma protein

*Mann-Whitney U-test; n represents the number of samples analyzed in each tumor type followed by the representative percentage for the total sample in the study; n.a. = Mann-Whitney U-test not applicable for n < 3.

Univariable statistical analysis showed a direct correlation between heparanase isoforms and the patient’s age. Since the average age of patients with gastrointestinal carcinoma is normally higher than the average age in the control group (the donors), Pearson’s correlation analysis was performed. The results clearly demonstrated that patients with different types of gastrointestinal carcinomas were older and presented higher expression of heparanase isoforms (heparanase-1 50 kDa: R = 0.658 and P < 0.0001; heparanase-1 65 kDa: R = 0.380 and P = 0.002; heparanase-2: R = 0.345 and P = 0.005), in comparison with the donor group (control).

In order to elucidate possible bias between older age and the presence of gastrointestinal carcinomas, multivariable statistical analysis was performed using logistic regression. The statistical parameters obtained through logistic regression proved that each heparanase isoform expression was straightforwardly dependent on the tumor [heparanase-1 (50 kDa): P < 0.0001; heparanase-1 (65 kDa): P = 0.002; and heparanase-2: P < 0.0001] and was independent of the patient’s age [heparanase-1 (50 kDa): P = 0.270; heparanase-1 (65 kDa): P = 0.218; and heparanase-2: P = 0.717].

Clinical feature correlations demonstrated that there was no significant difference between heparanase-1 (50 kDa), heparanase-1 (65 kDa) or heparanase-2 expression and tumor differentiation, tumor metastasis, tumor vascular invasion, lymphatic invasion or perineural infiltration, as observed through statistical analysis (data not shown).

### Detection of heparanase-1 enzymatic action on the plasma samples

**Figure 2.**
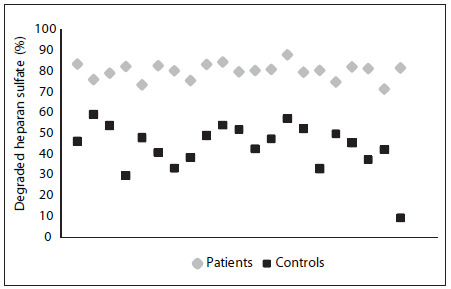
Analysis on plasma heparanase-1 activity by quantification of degraded heparan sulfate. Heparanase-1 enzymatic activity in plasma samples was measured by means of an ELISA-like method, using 15% biotinylated heparan sulfate immobilized in poly-L-lysine multiwall, as the substrate. The samples were obtained from plasma of gastrointestinal carcinoma patients (Patients) and healthy individuals (Controls). The values represent percentages of degraded heparan sulfate. The assay was performed in triplicate and repeated four times. There was a statistically significant difference between patients and controls (P < 0.0001).

The percentage of degraded heparan sulfate was used to quantify heparanase-1 enzymatic activity on the plasma samples. Figure 2 shows that there was significantly greater heparanase-1 enzymatic activity in the plasma of the gastrointestinal carcinoma patients (80.05%), compared with the control plasma (44.07%). Thus, the heparanase-1 enzyme activity in the plasma patients with gastrointestinal carcinoma was seen to be approximately twice as much as in the plasma of the unaffected individuals (Student’s t test, P < 0.0001).

### Plasma cathepsin B

**Figure 3.**
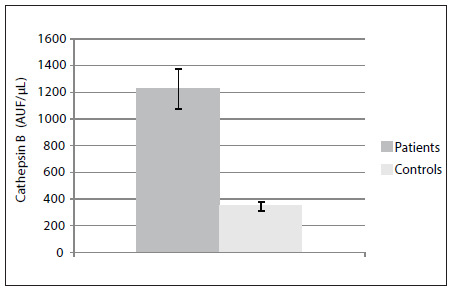
Cathepsin B activity was measured in plasma samples, using a fluorometric assay, as described in the Methods section. The values represent mean ± standard deviation. The assays were performed in triplicate and were expressed as arbitrary units of fluorescence (AUF) per µl of plasma. The plasma samples were collected from gastrointestinal carcinoma patients (Patients) and healthy individuals (Controls) (P < 0.0001).

The plasma cathepsin B enzymatic assay showed that its level was significantly higher in the patient group (1223 ± 147.2 AUF/µl) than in the control group (345 ± 32 AUF/µl), as demonstrated in Figure 3 (Student’s t test, P < 0.0001).

## DISCUSSION

In non-neoplastic human tissues, heparanase-1 mRNA is restricted to hematopoietic cells and placental cells, as described in the literature.^9^^,^^10^^,16^ In contrast, heparanase-2 expression is low in these tissues, but high in the brain, mammary gland, prostate, small intestine, testis, uterus and bladder tissues.^15^^,^^17^ This markedly different pattern of heparanase-2 expression suggests that, at least in normal tissues, the two heparanase isoforms may fulfill different functions.^19^ The results obtained in the present study demonstrated that heparanase-1 and heparanase-2 proteins were both present in the plasma.

Friedman et al. reported heparanase-1 tissue overexpression in cases of colon cancer progression and metastasis, among 16 patients with colon adenocarcinoma. They also concluded that the most poorly differentiated carcinoma tissues presented the highest expression of heparanase-1. High expression of heparanase-1 was also noted in other tissues like lung, lymph node and liver, as well as in the stromal tumor tissues. However, low heparanase-1 levels were observed in non-neoplastic tissues.^27^

The results obtained here showed that the level of the active form of heparanase-1 (50 kDa) was significantly higher in the plasma of the gastrointestinal carcinoma patients than in the control group. The greater enzymatic action of heparanase-1 in the plasma of the gastrointestinal carcinoma patients corroborates with the higher of protein expression found through Western blot analysis.

Furthermore, statistical analysis on the tumors grouped as different subtypes showed that the heparanase-1 (50 kDa) expression was significantly higher in all samples analyzed.

Cleavage of heparan sulfate by heparanase-1 (50 kDa) generated active oligosaccharides that were able to modulate cellular processes. Additionally, it was proved that these heparan sulfate oligosaccharides could increase the biological function of growth factors, angiogenic factors and cytokines, thus mediating cell proliferation, migration, inflammation and angiogenesis.^28^^,^^29^^,^^30^

It had already been observed in previous studies from our laboratory that heparanase-1 and heparanase-2 isoforms were overexpressed in the blood (mononuclear cell fraction) of women with breast cancer, thereby suggesting that this tumor can possibly modulate the expression of both heparanases.^31^ Similar results have been described, suggesting that higher heparanase expression in gastric cancer tissues was closely correlated with lower treatment responsiveness and poor prognosis.^32^

The data obtained in the present study demonstrated that heparanase-2 protein expression was significantly greater in plasma samples from patients with gastrointestinal carcinomas, compared with healthy individuals. Moreover, statistical analysis was also performed on different tumor type subgroups. It was observed that heparanase-2 levels were significantly higher in all subtypes of gastrointestinal carcinomas, compared with the control group.

Previous results from our group had shown that heparanase-2 was upregulated in all stages of colorectal carcinomas.^33^^,^^34^ Heparanase-2 may be involved in the carcinogenesis process through mediating tumor cell adhesion and migration or apoptosis, as shown by the latent form of heparanase-1 (65 kDa).^35^ Therefore, heparanase-2 may initiate signal transduction that activates proliferation and survival of colorectal carcinoma cells, independent of enzymatic activity.^33^^,^^34^ Nevertheless, further investigation is necessary, in order to validate this hypothesis concerning heparanase-2 function in colorectal carcinoma development. As suggested by Giordano, heparanase-2 upregulation in colorectal cancer may constitute a new marker for this neoplasia.^33^

Cathepsin B enzymatic activity was higher in the plasma samples of patients with gastrointestinal carcinomas than in the control group. Because cathepsin B cellular traffic can be modulated by the heparan sulfate proteoglycan cell surface, there may have been a correlation between cathepsin B and heparanase activity. In addition, cathepsin B seems to be localized in the perinuclear region of tumor cells and, consequently, the cellular distribution of heparan sulfate associated with growth factors may also have been altered. Therefore, the cathepsin B and heparan sulfate proteoglycan complex may play a role in nuclear functions, thereby becoming part of the transformation process that is observed in carcinogenesis.^22^^,^^26^

It is important to emphasize that the small number of samples for each type of gastrointestinal carcinoma may be a limitation to the present study. Nevertheless, the analysis on each subgroup demonstrated that there was a significant difference between the control and tumor samples.

The combined results obtained through analysis on heparanase and cathepsin B may have future implications for diagnosing gastrointestinal cancer. Heparanase and cathepsin B analysis may develop into a safer and less invasive novel diagnostic tool for gastrointestinal carcinomas.

## CONCLUSIONS

The analysis on heparanase isoforms and cathepsin B in the plasma of patients with gastrointestinal carcinomas that was proposed in the present study revealed that this is a potential new diagnostic tool.

This noninvasive assay for detecting different gastrointestinal carcinomas can be performed using plasma samples. The differences in plasma samples that were observed between the gastrointestinal carcinoma patients and unaffected individuals suggest that the protein expression of heparanase-1 (50 kDa) and heparanase-2 and also the enzymatic action of heparanase-1 and cathepsin B can possibly be used to detect the presence of carcinoma.

Further studies need to be undertaken in order to investigate whether the expression of heparanase isoforms and higher levels of enzymatic activity of heparanase-1 and cathepsin B in the plasma can be used to improve the diagnosis and also as potential novel therapeutic targets in cases of gastrointestinal carcinoma.

## References

[1] Cancer Research UK World cancer factsheet A3.

[2] Brasil. Ministério da Saúde. Instituto Nacional de Câncer Câncer de fígado.

[3] Arregi MMU, Férrer DPC, Assis ECV (2009). Perfil clínico-epidemiológico das neoplasias de estômago atendidas no Hospital do Câncer do Instituto do Câncer do Ceará, no período 2000-2004 [Clinical-epidemiological profile of gastric neoplasms in the cancer Hostpital of Ceara’s Cancer Institute, from the period 2000-2004]. Rev Bras Cancerol.

[4] Wakatsuki T, Irisawa A, Takagi T (2008). Primary adenocarcinoma of the minor duodenal papilla. Yonsei Med J.

[5] Devuni D, Birk JW Papillary tumors. Medscape.

[6] National Cancer Institute What You Need To Know About™ Cancer of the Colon and Rectum.

[7] Queiroga RC, Pernambuco AP (2005). Câncer de esôfago: epidemiologia, diagnóstico e tratamento [Esophageal cancer: epidemiology, diagnosis and treatment]. Rev Bras Cancerol.

[8] Chen JQ, Zhan WH, He YL (2004). Expression of heparanase gene, CD44v6, MMP-7 and nm23 protein and their relationship with the invasion and metastasis of gastric carcinomas. World J Gastroenterol.

[9] Vlodavsky I, Friedmann Y, Elkin M (1999). Mammalian heparanase: gene cloning, expression and function in tumor progression and metastasis. Nat Med.

[10] Hulett MD, Freeman C, Hamdorf BJ (1999). Cloning of mammalian heparanase, an important enzyme in tumor invasion and metastasis. Nat Med.

[11] Naomoto Y, Takaoka M, Okawa T (2005). The role of heparanase in gastrointestinal cancer (Review). Oncol Rep.

[12] Vlodavsky I, Eldor A, Haimovitz-Friedman A (1992). Expression of heparanase by platelets and circulating cells of the immune system: possible involvement in diapedesis and extravasation. Invasion Metastasis.

[13] Vlodavsky I, Goldshmidt O, Zcharia E (2001). Molecular properties and involvement of heparanase in cancer progression and normal development. Biochimie.

[14] McKenzie E, Tyson K, Stamps A (2000). Cloning and expression profiling of Hpa2, a novel mammalian heparanase family member. Biochem Biophys Res Commun.

[15] Baker E, Crawford J, Sutherland GR (1999). Human HPA endoglycosidase heparanase. Map position 4q21.3. Chromosome Res.

[16] Vlodavsky I, Goldshmidt O (2001). Properties and function of heparanase in cancer metastasis and angiogenesis. Haemostasis.

[17] Dempsey LA, Brunn GJ, Platt JL (2000). Heparanase, a potential regulator of cell-matrix interactions. Trends Biochem Sci.

[18] Marchetti D, Li J, Shen R (2000). Astrocytes contribute to the brain-metastatic specificity of melanoma cells by producing heparanase. Cancer Res.

[19] Goldshmidt O, Zcharia E, Cohen M (2003). Heparanase mediates cell adhesion independent of its enzymatic activity. FASEB J.

[21] Nadav L, Eldor A, Yacoby-Zeevi O (2002). Activation, processing and trafficking of extracellular heparanase by primary human fibroblasts. J Cell Sci.

[21] Levy-Adam F, Feld S, Cohen-Kaplan V (2010). Heparanase 2 interacts with heparan sulfate with high affinity and inhibits heparanase activity. J Biol Chem.

[22] Yan S, Sameni M, Sloane BF (1998). Cathepsin B and human tumor progression. Biol Chem.

[23] Eldor A, Bar-Ner M, Yahalom J, Fuks Z, Vlodavsky I (1987). Role of heparanase in platelet and tumor cell interactions with the subendothelial extracellular matrix. Semin Thromb Hemost.

[24] Ishai-Michaeli R, Eldor A, Vlodavsky I (1990). Heparanase activity expressed by platelets, neutrophils, and lymphoma cells releases active fibroblast growth factor from extracellular matrix. Cell Regul.

[25] Bouças RI, Trindade ES, Tersariol IL, Dietrich CP, Nader HB (2008). Development of an enzyme-linked immunosorbent assay (ELISA)-like fluorescence assay to investigate the interactions of glycosaminoglycans to cells. Anal Chim Acta.

[26] Almeida PC, Nantes IL, Chagas JR (2001). Cathepsin B activity regulation. Heparin-like glycosaminoglycans protect human cathepsin B from alkaline pH-induced inactivation. J Biol Chem.

[27] Friedmann Y, Vlodavsky I, Aingorn H (2000). Expression of heparanase in normal, dysplastic, and neoplastic human colonic mucosa and stroma. Evidence for its role in colonic tumorigenesis. Am J Pathol.

[28] Dreyfuss JL, Regatieri CV, Jarrouge TR (2009). Heparan sulfate proteoglycans: structure, protein interactions and cell signaling. An Acad Bras Cienc.

[29] Sanderson RD, Yang Y, Kelly T (2005). Enzymatic remodeling of heparan sulfate proteoglycans within the tumor microenvironment: growth regulation and the prospect of new cancer therapies. J Cell Biochem.

[30] Vreys V, David G (2007). Mammalian heparanase: what is the message?. J Cell Mol Med.

[31] Theodoro TR, de Matos LL, SantAnna AV (2007). Heparanase expression in circulating lymphocytes of breast cancer patients depends on the presence of the primary tumor and/or systemic metastasis. Neoplasia.

[32] Takaoka M, Naomoto Y, Ohkawa T (2003). Heparanase expression correlates with invasion and poor prognosis in gastric cancers. Lab Invest.

[33] Giordano RJ (2008). Heparanase-2 and syndecan-1 in colon cancer: the ugly ducklings or the beautiful swans?. Eur J Gastroenterol Hepatol.

[34] Peretti T, Waisberg J, Mader AM (2008). Heparanase-2, syndecan-1, and extracellular matrix remodeling in colorectal carcinoma. Eur J Gastroenterol Hepatol.

[35] Marques RM, Focchi GR, Theodoro TR (2012). The immunoexpression of heparanase 2 in normal epithelium, intraepithelial, and invasive squamous neoplasia of the cervix. J Low Genit Tract Dis.

